# Treating Intertrochanteric Fracture by Short and Long Cephalomedullary Nail: A Systematic Review and Meta-analysis

**DOI:** 10.5704/MOJ.2603.001

**Published:** 2026-03

**Authors:** QN Erman, MN Norhayati, TM Shihabudin

**Affiliations:** 1 Department of Orthopaedics, Universiti Sains Malaysia, Kubang Kerian, Malaysia; 2 Department of Family Medicine, Universiti Sains Malaysia, Kubang Kerian, Malaysia

**Keywords:** long nail, short nail, pertrochanteric, proximal femur fracture, intratrochantric

## Abstract

Intertrochanteric femur fracture is a common injury, and cephalomedullary nailing has become a popular surgical technique for the treatment of these types of fracture patterns. Various nail implant designs exist, including both short and long versions. The initial design of the implant was a short cephalomedullary nail, but it was associated with problems such as increased hip pain and periprosthetic fracture. To address these issues, a longer nail was developed. In this review, we determine the advantage of treating the intertrochanteric fracture using long and short intramedullary nails. We also compared the outcomes among the different types of nail manufacturers. All trials comparing long and short nails were included. We searched Google Scholar, PubMed, central Cochrane, Clinic Trials and Science Direct. Two authors screened and reviewed studies independently and collected data using fixed-effect models. The results were presented as risk ratio (RR) and mean difference (MD) at 95% confidence intervals (CI). Twenty trials were included with a total of 3470 patients. The results showed that the short nail group had a shorter operative time, less blood loss and hip pain, lower transfusion and peri-implant fracture rate. Functional outcomes were favourable for both nail types, though scores were slightly better in the long nail group. There was no significant difference in mortality, complication rates, or reoperation rates between the two groups.

## Introduction

Trochanteric fracture is defined as any fracture between the greater trochanteric attachment for gluteus medius and gluteus minimus (hip extensor and abductors) and the lesser trochanter where the iliopsoas are attached. Almost nine out of ten hip fractures occur in patients older than 65 years old, while three out of four fractures occur in women ^1^. The rate of unstable and comminuted fractures is increasing, corresponding to the increase in the lifespan of the world’s population ^2^.

The fixation method can be achieved using an extramedullary or intramedullary device. Currently, the proximal femoral nail has become a very popular intramedullary device used by surgeons worldwide to treat proximal femur fractures, as it has several mechanical advantages over extramedullary devices ^3^.

Initially, the proximal femoral nail was short. The longer version of the nail was introduced to address concerns about stress riser forming at the tip of this short nail that may subsequently lead to fracture at this region. It was reported that the original Gamma nail (Howmedica International, London, UK) was associated with femoral shaft fracture ranging from 6 - 17% ^4-8^. In addition, there were concerns that long nails might increase the risk of anterior cortex fractures.

However, with the emergence of newer versions of nails, several studies showed this problem had become less significant ^9^. There are several benefits that some surgeons thought would favour the usage of long nails, such as protecting the full length of the femur, particularly for elderly patients who suffer from osteoporotic bone. On the other hand, some studies suggest that short nails may result in less blood loss, shorter operative time, and reduced need for blood transfusion^3,9^.

Several clinical studies have compared the outcomes of the short proximal intramedullary nail and the long nail. Both designs have several advantages and disadvantages. Most of these studies were retrospective ^9^. Only recently have a few studies prospectively compared the short and long nails head-to-head. Additionally, a limited number of studies have evaluated the biomechanics of cephalomedullary nails ^10-12^.

We reviewed studies that evaluated the advantages of short and long nails and assessed outcomes across various nail designs from different manufacturers. The information would be helpful to surgeons treating this fracture in choosing the appropriate implant.

## Review

This research was conducted in accordance with the Preferred Reporting Items for Systematic Reviews and Meta-Analyses (PRISMA) guidelines. The registration number is CRD42021236720. Additionally, the research protocol was registered with the International Prospective Register of Systematic Reviews (PROSPERO).

### Data Sources and Searches

The search was confined to the databases of the Web of Science (WOS), Science Direct, PubMed, and Google Scholar that were published until March 2021. The keywords used were Intertrochanteric, Extracapsular, and Pertrochanteric fractures, Intramedullary and Cephalomedullary nails, Proximal femur nail, Gamma nail, InterTAN nail, and long and short nails.

### Eligibility Criteria

All randomized controlled trials and retrospective cohort studies comparing long and short intramedullary nails for the treatment of intertrochanteric fractures were included. Only publications in English were considered. Each study was required to report at least one measurable outcome, such as functional results, blood transfusion requirements, length of hospital stay, mortality, operative time, volume of blood loss, postoperative complications, or implant failure.

### Trial Selection

The titles and abstracts were identified from the searches, and full-text articles were obtained when they appeared to meet the eligibility criteria or when there was insufficient information to assess the eligibility. The trials' eligibility was independently evaluated, and the reasons for exclusion were documented. Any disagreements between the review authors were resolved by discussion, and the authors were contacted if clarification was needed.

### Data Extraction

Using a data extraction form, we extracted the study setting, participant characteristics (age, sex, ethnicity), methodology (number of participants randomised and analysed, duration of follow-up), types of nails, and types of fractures from each of the selected trials.

### Risk of Bias Assessment

Risk of bias was assessed based on several criteria: random sequence generation, allocation concealment, blinding of participants and personnel, blinding of outcome assessors, completeness of outcome data, selective outcome reporting, and other potential sources of bias. Any disagreements between reviewers were resolved through discussion.

### Data Synthesis

Meta-analyses were conducted using Review Manager 5.4 software (RevMan 2020), if appropriate, and a random-effects model was used to pool data, depending on the degree of significant clinical or statistical heterogeneity. Thresholds for interpreting the I2 statistic can be misleading since the importance of inconsistency depends on several factors. We followed standard guidelines for interpreting heterogeneity as outlined: 0% to 40% might not be important, 30% to 60% may represent moderate heterogeneity, 50% to 90% may represent substantial heterogeneity, and 75% to 100% have considerable heterogeneity.

## Results

### Study Selection

**Fig. 1:**
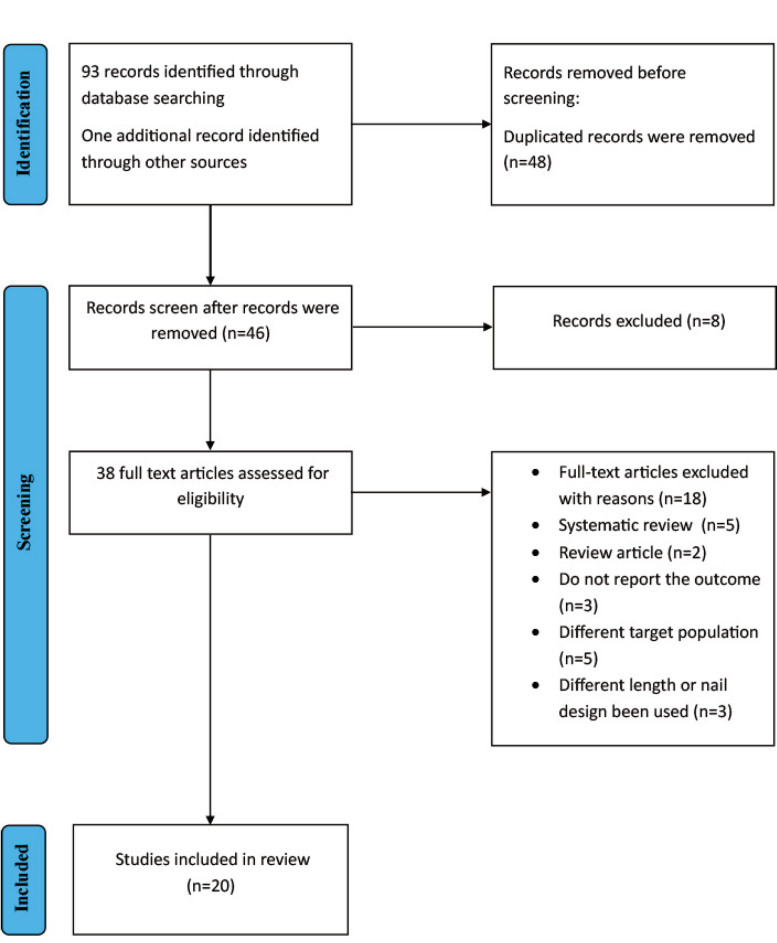
Study PRISMA flow diagram.

We identified 93 records through database searching and one additional record from other sources (Fig. 1), resulting in a total of 94 records screened. After reviewing the full texts of 38 studies, 18 papers were excluded based on eligibility criteria.

Out of the 18 non-eligible papers, five were systematic reviews that were not related to our research question ^3,13-16^, two papers were review articles ^9,17^, three papers did not report the outcome ^18-20^, two papers had a different target population ^21,22^ (one focused on revision cases previously done intertrochanteric fracture fixation using intramedullary nail, the other only compared cases that had peri-implant fracture). Additionally, three studies involved variations in nail length or design that did not meet our inclusion criteria ^23-25^ (one paper unspecified which generation of short they used first or second, the other paper compared two short nails, and the last study the outcome of intermediate nails). Therefore, we finally included 20 articles in our study (Table I).

**Table I:** Characteristics of included studies.

Studies	Country	Study design	Types of nails	Follow-up (year)	Age distribution	Sex distribution	Number of patients: Long and short nails
Boone et al^26^, 2014	Michigan, USA	Retrospective study	Gamma3	Minimum 1 year	81+/- 9.2	Female: 140, Male: 54	Short: 82, Long: 119
Frisch et al ^27^, 2016	Detroit, USA	Retrospective study	Inter Tan	At least 8 weeks	Short: 76.2 ± 12.3 Long: 76.3 ± 15.2	Female: 121, Male: 48	Short: 72, Long: 97
Galanopoulos et al ^28^, 2018	Athens, Greece	Prospective study	The Affixus Hip Fracture Nail System, Orthofix VeroNail, Trochanteric Nail	Follow-up 2 years (range 1 - 5)	74-93	Female: 33, Males: 17	Short: 25, Long: 25
Guo et al^29^, 201 5	Tianjin, China	Retrospective study	Gamma3	1 - 2 years	65-89	Female: 93 Male: 85	Short: 102, Long: 78
Hari Krishnan et al^30^, 2019	Uttar, India	Retrospective study	PFNA	Minimum 1 year	75 (60 - 90)	Female: 106, Male: 64	Short: 81, Long: 89
Hong et al^31^, 2017	Singapore	Retrospective study	PFNA	Minimum 1 year	Short: 80 (60 - 93) Long: 79.8 (56- 97)	Female: 45, Male: 19	Short: 44, Long: 20
Hou etal^32^, 201 3	Hebei, China	Retrospective study	TFN	Minimum 1 year	79 (47 - 102)	Female: 210, Male: 73	Short: 100, Long: 183
Kleweno et al^33^, 2014	Boston USA	Retrospective study	Gamma 2/3, TFN	Minimum 1 year	84 (65 - 102)	Female: 404, Male: 155	Short: 219, Long: 340
Krigbaum et al^34^, 2016	San Francisco, USA	Retrospective study	TFN, Gamma nail. Intertan nail	Short nail, mean 2.8 year; Long nail mean 2.2 year	60 and above	Male: 262	Short: 125, Long: 137
Mahesh Kumar et al^35^, 2017	India	Prospective randomised PFN comparative study	PFN	1 year	Above 50	80 subjects (sex not stated)	Short: 40, Long: 40
Li etal^36^, 2015	Shijiazhuang, China	Retrospective study	PFNA	Minimum 1 year	Short: 76.81 ± 6.56 Long: 74.85 ± 8.15	Female: 90, Male: 66	Short: 97, Long: 59
Shyamkumar et al^37^, 2018	Vijayawada, India	Prospective study	PFN	Minimum 6 months	Short: 75.3 Long: 73.7	Female: 11 Male: 19	Short: 15 Long: 15
Lindvall et al^38^, 201 6	Fresno, USA	Retrospective study	PFNA, TFN , Gamma3	Minimum 1 year	Short: 71.9 (18-97) Long: 73.0 (13 - 105)	Female: 360, Male: 249	Short: 171, Long: 438
Okcu et al^39^, 2013	Turkey	Prospective study	PFNA	Minimum 1 year	79 (67 - 95)	Female: 25, Male: 8	Short: 15, Long: 18
Parmar et al^40^, 2011	Himmatnagar, India	Retrospective study	PFNA	Minimum 6 months	Short: Average 60 Long: Average 62	Female: 69, Male: 55	Short: 52, Long: 72
Raval et al^41^, 2016	Scotland, United Kingdom	Retrospective study	PFNA	Minimum 1 year	Short: 77 (68 - 86) Long: 76 (68 - 84)	Female: 57, Male: 23	Short: 40, Long: 40
Sellan et al^42^, 2019	London, United Kingdom	Prospective study	Intertan	Minimum 1 year	79 (range: 56- 97)	Female: 75, Male: 35	Short: 71, Long: 39
Shannon et al^43^, 2019	USA	Prospective study	TFN, Gamma3, Affixus	Mean 13.9 months. Minimum 3 months Long nail: 79	Short nail: 82 (79 - 84)	Female: 74, Male: 34	Short: 80, Long: 88
Thamyongit et al^44^, 2020	Baltimore, USA	Retrospective study	Unidentified nail	Minimum 6 months	76 ± 15	Female: 22, Male: 21	Short: 18, Long: 25
Vaughn et al^45^, 201 5	Rhode Island, USA	Retrospective study	Gamma3	Minimum 1 year	NA	NA	Short: 60, Long: 196

### Participants

Twenty studies were included, comprising a total of 3,470 patients ^26-45^ (Table I). Ten of the these studies were conducted in high-income countries ^26,27,33,34,38,41-45^, while the other ten were in middle-and low-income countries ^28-32,35-37,39,40^. Of the twenty studies, ten were carried out in university hospitals ^28,31,32,34-36,38,39,42,44^. One trial recruited participants from a non-profit clinic ^43^, and the rest recruited participants from trauma centres (general hospitals).

### Quality Assessment

In terms of risk of bias assessment, our trials noted a generally unclear to lower risk of predominant bias. However, since most of our data was retrospectively retrieved and contained studies with a lack of full control, this resulted in an unclear risk of bias in our selective bias. There was no report of attrition bias, while only two studies had a high risk of reporting bias.

### Outcome

The primary outcome, i.e., the function outcome, has been reported in eight trials ^28,35-37,39,42-44^. However, only four were included in the meta-analysis, each with a minimum follow-up period of three months.

Out of the four excluded studies, one trial used figures to present their result ^42^, while one trial mentioned functional outcomes as there was a similarity between the two groups without any data ^28^. The remaining two studies lacked data for SD calculation, and the authors could not be contacted ^35,37^. Secondary outcomes were reported in all. Each outcome and the trial included are stated in the results section.

### Function outcome

Four out of eight studies used a Harris Hip Score ^36,39,43,44^, while in the other four studies, SD was incalculable due to a lack of data or because they used different types of scores, such as the Extremity Functionality scale or the Functional Independence measure.

**Fig. 2:**
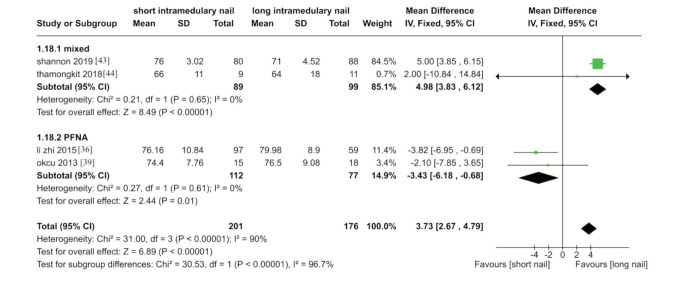
Meta-analysis of functional outcome after fixation with short vs long intramedullary devices.

These four trials used different types of nails, and two of them reported functional outcomes after one year ^36,39^. One trial calculated the function outcome at three months of follow-up ^43^, while another one at six months of follow-up ^44^. Our analysis showed that functional outcomes were better with long nails (Fig. 2).

However, Li Z *et al* ^36^ and Okcu *et al* ^39^ found function outcome better in short, and both of the studies used proximal femoral nail antirotation [PFNA; Synthes GmbH, Oberdorf, Switzerland] (MD 3.73, 95% CI 2.67-4.79, I^2^statistic =90%; P<0.001; four trials, 377 participants; moderate quality evidence).

### Blood Loss and Transfusion

Nine studies analysed blood loss ^26,27,29,30,32,36,41,43,44^ with a range of 20 to 500ml in the short nail group and 20 to 1000ml in the long nail group. Our results showed a significant reduction of blood loss in short nails, which is considered a simpler procedure with fewer steps, and it rarely required reaming and easier distal screw insertion as compared to long nails (MD -74.80, 95%CI -79.85-- 69.74, I^2^= 98%; P<0.001; 9 trials, 1448 participants, moderate quality evidence).

**Fig. 3:**
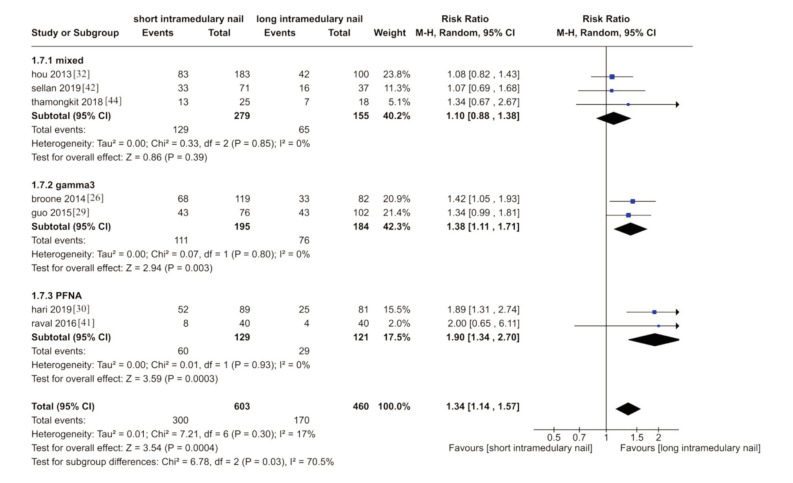
Meta-analysis of blood transfusion after fixation with short vs long intramedullary devices.

Blood transfusion was discussed in seven studies ^26,29,30,32,41,42,44^. These studies showed that more blood transfusion was needed in the long nail group. (RR 1.34, 95% CI 1.17-1.55; I^2^= 70.5; P=0.03; 7 trials, 1065 participant; moderate quality evidence) (Fig. 3).

### Duration of Surgery

**Fig. 4:**
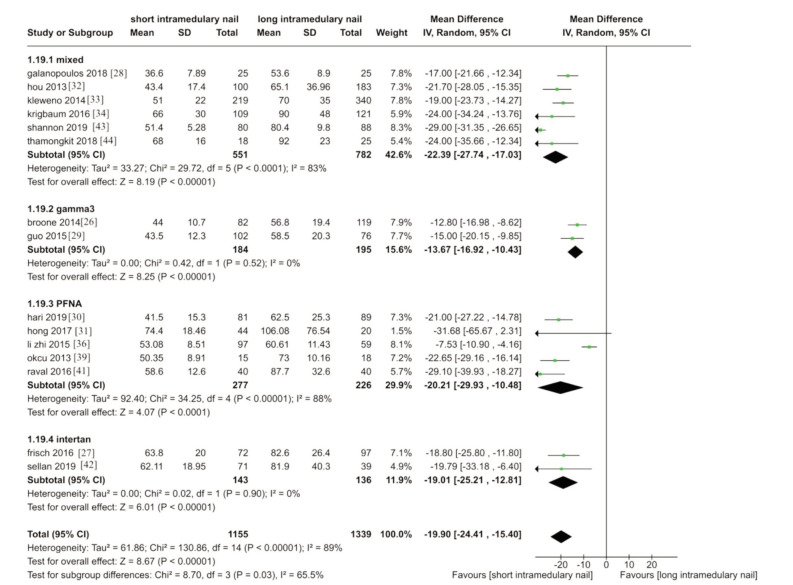
Meta-analysis of duration of surgery during fixation using short vs long intramedullary devices.

A total of 18 trials measured operative time26-36,38-44. Three were excluded due to insufficient data to calculate SD [35, 38, 40]. The results showed reduced operative time in short nails (Fig. 4). (MD -19.90, 95%CI -21.41 - -15.40, I^2^= 89%; P <0.001; 15 trials, 2494 participants, moderate quality evidence).

### Length of Hospital Stay

**Fig. 5:**
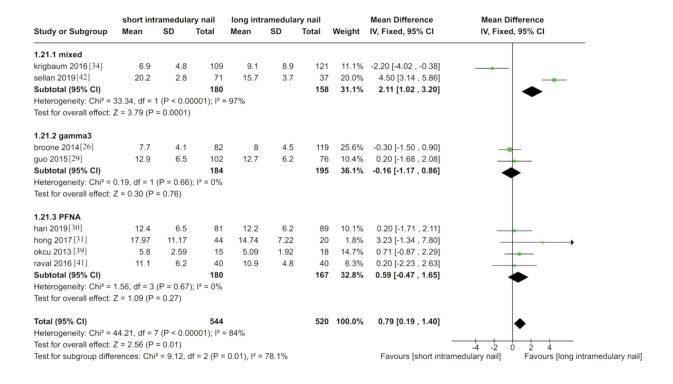
Meta-analysis of length of hospital stay after fixation with short vs long intramedullary devices.

Results showed an increase in the length of stay for patients treated with long nails (Fig. 5). The subgroup study showed no difference between the two groups of studies that used gamma3 and PFNA. However, the results showed an increase in hospital stays in the long nail group in the study with mixed nails. Ten trials mentioned this outcome, out of which eight were used for this meta-analysis ^26,29,31,34,39,41,42^. Two trials were excluded here due to a lack of data to calculate SD (MD 0.79, 95%CI 0.19-1.40, I^2^= 84%; P<0.001; 8 trials, 1064 participants; moderate quality evidence).

### Peri-implant Fracture

**Fig. 6:**
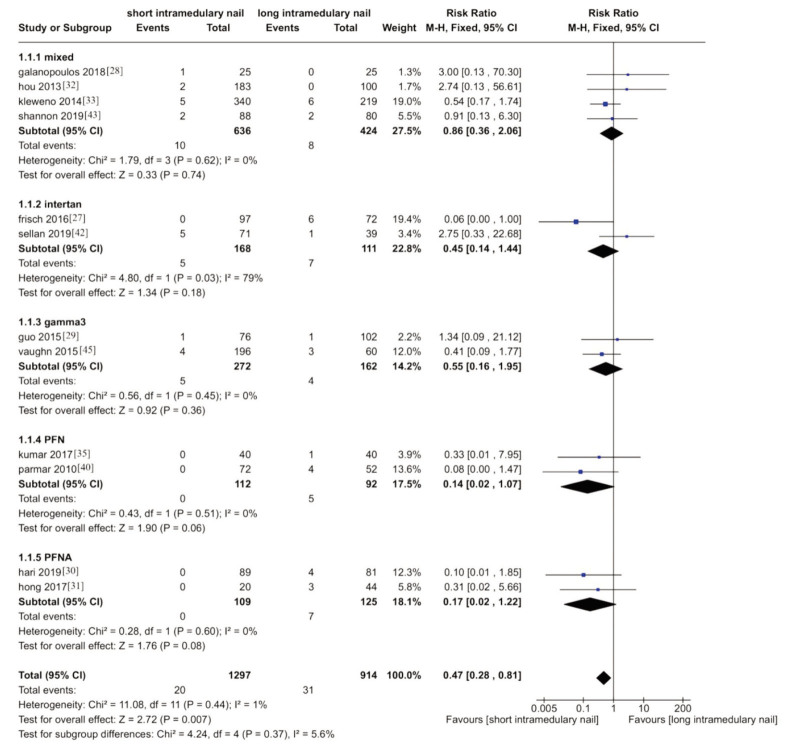
Meta-analysis of peri-implant fracture after fixation with short vs long intramedullary devices

Thirteen trials recorded the accident of peri-implant fracture, of which 12 were included in our meta-analysis ^27-33,35,40,42,43,45^. One trial was excluded as it recorded zero accidents in both groups (RR 0.47, 95%CI 0.28-.81, I^2^= 1%, P=0.44; 12 trials, 2252 participants, moderate quality evidence). Results showed slightly lower accidents in the short nail group, while the results in the subgroup study showed no difference between groups with trials using Gamma3 (Stryker) nails (Fig. 6).

However, trials that used the InterTAN [IT; Smith & Nephew Richards, Memphis, TN], Proximal Femoral Nail [PFN; Synthes GmbH, Oberdorf, Switzerland], and PFNA reported a higher incidence of complications in the short nail group.

### Hip Pain

**Fig. 7:**
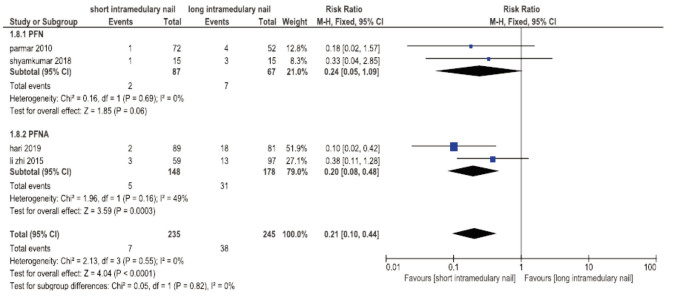
Meta-analysis of hip pain after fixation with short vs long intramedullary devices.

Four trials have followed-up patients to evaluate the hip pain or anterior thigh pain30,36-37,40 and the result showed a lower incidence of hip pain in the short nail group (RR 0.21, 95%CI 0.10-0.44, I^2^= 0%; P=0.55; 4 trials, 480 participants, low quality evidence) (Fig. 7).

### Mortality

**Fig. 8:**
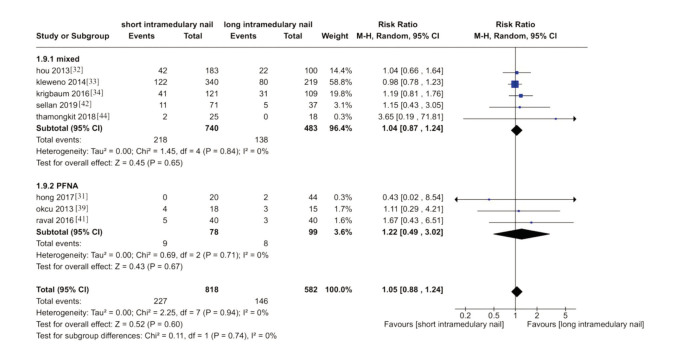
Meta-analysis of mortality after fixation with short vs long intramedullary devices

No difference between the two groups was reported (Fig. 8). Eight trials reported the numbers of mortality encounters during trials ^31-34,39,41,42,44^. (RR1.05, 95%CI 0.88-1.24, I^2^= 0%; P=0.94; 8 trials, 1402 participant; moderate quality evidence).

### Post-operative Complications

We analysed different kinds of complications that include union and ambulation status, re-operative rate, screw cutout, infection, avascular necrosis, and heterotopic ossification. No difference was found between the two groups except for hip pain. Results reported fewer hip pain accidents in the short nail group.

## Discussion

Since the introduction of intramedullary nails (IMNs) for treating proximal femoral fractures, nail designs have changed to address previously reported complications. A proximal femoral nail is considered short if it does not extend beyond the femoral isthmus, whereas nails that cross the isthmus and measure longer than 250 mm are classified as long nails ^46^. Currently, not only do we have the new generation of short and long nails, but the overall design of the nail also differs according to the manufacturers. Our study analysed the outcomes of these different nail designs and lengths.

When nail designs from different manufacturers were grouped into short and long categories, we found that functional outcome scores were higher in the long nail group. Our analysis came from four studies that utilised the Harris hip score for the outcome parameter. However, these studies only assessed the score in the early post-operative phase. Two studies evaluate at one year, and another two studies assess at three to six months. We believe this assessment period may be too early to draw definitive conclusions about long-term functional recovery.

Interestingly, when these nails were divided into types of nail manufacturers that were gamma [Stryker Orthopaedics, Mawah, NJ], PFNA, and Trochanteric Femoral Nail [TFN; Synthes, Paoli, PA], the results showed that the short PFNA design demonstrated better functional outcomes compared to long PFNA. However, long gamma and long TFN showed better functional outcomes than the short nail. We noted a randomised prospective study by Dragosloveanu *et al* ^47^, which stated that the short nail has a significantly better Harris Hip score at six months post-operative when compared to the long nail. However, the outcome was similar at one year between short and long nails. Only the short and long 3rd-generation gamma nails were compared in their study. These findings suggest that the newer generation of short nails is not inferior to long nails.

Long nails are believed to protect the whole bone, which reduces the incidence of fractures, but the change of design of short nails over time has helped to improve it and reduce the chances of fractures. Tan *et al* ^26^ noted a significant reduction of peri-implant fracture rate in the long nail group, but the authors did not specify which nail generation was involved in their study. We suspect the study involved the old and new generations of nails, which made the results differ. Norris *et al* ^48^ showed that in the newer generation of nails, the short nail had no significant increase in peri-prostatic fracture rate, but there was a performance improvement in the group treated with long nails. The same findings were observed in our study, as there was a better Harris hip score with long nails and a similar periprostatic fracture rate.

A meta-analysis by Zang *et al* ^13^, which also examined newer-generation nails, found comparable reoperation and periprosthetic fracture rates between short and long nail groups. Similarly, a recently published large cohort study by Larose *et al* ^49^ evaluated 970 patients with intertrochanteric fractures treated using either short or long nails and reported no significant difference in periprosthetic fracture rates between the two groups. These findings are consistent with our analysis.

Hip pain or thigh pain is thought to be related to a short nail due to the impingement of the nail tip to the anterior femoral cortex. However, Dubey *et al* ^50^ concluded that anterior thigh pain is not inherently linked to short nails and may be avoided with proper surgical technique. Our review supports the advantage of short nails over long nails in reducing hip and anterior thigh pain, as four studies evaluating this parameter reported favourable outcomes with short nails. None of the reviewed articles assessed knee pain in relation to nail length.

Many findings in our study align with those of previous research ^13,47,51,52^. These are increased blood loss, transfusion rate, and operative time in the long nail group. Therefore, in the light of the new generation of short nails that are not inferior to long nails, the surgeon should consider the usage of short nails, especially when operating time, blood loss, or blood transfusion become important factors in managing the cases.

Apart from short and long intramedullary nails, the design of the nail differs depending on the nail manufacturer. Cheng and Sheng *et al* ^52^, in their meta-analysis study, evaluated different methods of intertrochanteric fracture fixation using both extramedullary and intramedullary fixation devices. The authors found that PFNA is the optimal treatment for intertrochanteric fixation and achieved the highest score, but did not mention the outcome with the short or long nail.

There is always a concern in elderly patients, as the bone quality may be affected. Nasim *et al* ^53^ reviewed 999 patients in a single-centre cohort study and concluded that in a predominantly female cohort aged over 75 years, the risk of periprosthetic fracture and implant failure was similar between short and long nail groups for intertrochanteric fractures. Unfortunately, our review could not analyse the influence of gender or bone quality on outcomes, as none of the twenty included studies provided adequate comparative data on these aspects. This represents a limitation of our review.

No comparison was made to determine whether fracture patterns influenced the the outcomes of short versus long nails. Most of the twenty studies we reviewed classified fractures using the AO/OTA Type 31 system, which includes subtypes A1, A2, and A3. Further research exploring the relationship between specific fracture patterns and nail length would be beneficial.

Overall, the heterogeneity was low; however, we have high heterogeneity in functional outcome (I2=90%). This can be explained based on the type of nail being used in addition to blood loss (I2=98, blood transfusion (I2=70.5), duration of surgery (I2=89%), and the wide range of differences in surgeon skills. We also noticed high heterogeneity in the length of hospital stay (I2=84%), which could be related to different comorbidities in each patient.

### CONCLUSION

The use of short nails in the treatment of intertrochanteric fractures was associated with lesser blood loss and transfusion, duration of surgery, hospital stays, and lower rates of peri-implant fractures and anterior thigh or hip pain. Short nail also has a comparable outcome to long nail regarding mortality rate and post-operative complications. Overall, the functional outcomes were good for both nails, but the scores, however, were better with the use of long nail.
